# A Multicomponent Approach to Identify Predictors of Hospital Outcomes in Older In-Patients: A Multicentre, Observational Study

**DOI:** 10.1371/journal.pone.0115413

**Published:** 2014-12-26

**Authors:** Stefanie L. De Buyser, Mirko Petrovic, Youri E. Taes, Davide L. Vetrano, Graziano Onder

**Affiliations:** 1 Department of Geriatrics, Ghent University Hospital, Ghent, Belgium; 2 Department of Endocrinology and Unit for Osteoporosis and Metabolic Bone Diseases, Ghent University Hospital, Ghent, Belgium; 3 Centro Medicina dell’Invecchiamento, Università Cattolica del Sacro Cuore, Rome, Italy; Federal University of Rio de Janeiro, Brazil

## Abstract

**Background:**

The identification of older patients at risk of poor hospital outcomes (e.g. longer hospital stay, in-hospital mortality, and institutionalisation) is important to provide an effective healthcare service.

**Objective:**

To identify factors related to older patients’ clinical, nutritional, functional and socio-demographic profiles at admission to an acute care ward that can predict poor hospital outcomes.

**Design and Setting:**

The CRiteria to assess appropriate Medication use among Elderly complex patients project was a multicentre, observational study performed in geriatric and internal medicine acute care wards of seven Italian hospitals.

**Subjects:**

One thousand one hundred twenty-three consecutively admitted patients aged 65 years or older.

**Methods:**

Hospital outcomes were length of stay, in-hospital mortality, and institutionalisation.

**Results:**

Mean age of participants was 81 years, 56% were women. Median length of stay was 10 (7–14) days, 41 patients died during hospital stay and 37 were newly institutionalised. Number of drugs before admission, metastasized cancer, renal failure or dialysis, infection, falls at home during the last year, pain, and walking speed were independent predictors of LoS. Total dependency in activities of daily living and inability to perform grip strength test were independent predictors of in-hospital mortality. Malnutrition and total dependency in activities of daily living were independent predictors of institutionalisation.

**Conclusions:**

Our results confirm that not only diseases, but also multifaceted aspects of ageing such as physical function and malnutrition are strong predictors of hospital outcomes and suggest that these variables should be systematically recorded.

## Introduction

The identification of geriatric patients at risk of poor hospital outcomes (e.g. longer hospital stay, in-hospital mortality, institutionalisation) is important to provide an effective health care service [1]. Predicting hospital outcomes at admission may facilitate the healthcare organisation, as it allows staff to optimally manage healthcare resources. Additionally, providing information regarding prognosis may help the individual patient in terms of planning care [1]–[4].

Most studies rely on routinely collected data such as age, gender, and clinical diagnosis when looking at outcomes of hospitalised older adults. However, a systematic literature review by Campbell *et al.* [5] underlined the need to take into account multifaceted aspects such as nutritional and functional status. Indeed, these aspects are rarely considered in clinical research. For example, very few studies performed in the acute care setting have included physical performance measures. Nevertheless these measures cover multiple facets and have been shown to predict hospital outcomes [2], [6], [7].

The aim of the present study was to identify which factors on admission can predict hospital outcomes in older patients admitted to an acute care ward. In pursuit of this objective, we used a multi-component approach. First, we addressed multiple hospital outcomes, i.e. length of hospital stay (LoS), in-hospital mortality, and institutionalisation. Second, we analysed prospectively collected data, including both *socio-demographic* factors and *multifaceted* aspects of ageing e.g. nutritional status, functional status and physical performance measurements.

## Methods

### Ethics statement

The study complies with the ethical rules for human experimentation that are stated in the Declaration of Helsinki. All participating hospitals had obtained approval for the study from their ethical committee. Written, informed consent was obtained from either the patient or the surrogate legal representative.

### Study population

The Criteria to assess appropriate Medication use among Elderly complex patients (CRIME) study was a multicentre, observational study, aimed to assess prescribing patterns in older adults hospitalised in Italy and to produce recommendations for pharmacological prescribing in older complex patients. Details about these results of the CRIME project are reported elsewhere [8]–[12]. Researchers of three academic hospitals in Italy (Università Cattolica in Rome, University of Perugia, University of Ferrara) and researchers of four centres of the Italian National Institute of Health and Science on Aging (INRCA)(situated in Ancona, Cosenza, Fermo, and Rome) recruited patients, who were consecutively admitted to geriatric and internal medicine acute care wards. Age of at least 65 years and willingness to participate were the only inclusion criteria. Between June 2010 and May 2011, 1123 hospitalised older in-patients were enrolled in the study.

### Data collection

A questionnaire was designed to assess the participants at admission and at daily intervals until discharge. Study researchers had received a two-day training course in which they were well-instructed about how to correctly collect and report questionnaire data. The researchers were ordinary clinical staff, external research staff, or a combination of both. They used a variety of information sources, including direct observation, clinical records, and interviews with the patients, family, friends or formal service providers.

Patient-related data included socio-demographic factors and medical history. Type of admission was classified into admission through the emergency room and elective admission (if previously planned). Comorbidity score was the count of a set of 50 medical diagnoses comprising cardiovascular, endocrine, genitourinary, musculoskeletal, neurological, and malignant diseases at admission or hospital-acquired. Additionally, presence of clinical conditions (falls at home during the last year, pain, pressure ulcers, incontinence) was reported. Furthermore, nutritional status (body mass index (BMI)), cognitive status (30 item Mini Mental State Examination (MMSE)[13]), and affective status (15 item Geriatric Depression Scale [14]) were evaluated.

Dependency in six activities of daily living (ADLs) [15] (transferring, bathing, dressing, eating, bowel and bladder continence, and personal hygiene) was reported to assess functional status. Walking speed was assessed by having the participant walk at his/her usual pace over a four-meter course. This test has shown a high test-retest reliability [16]. Grip strength was measured using a North Coast Medical hand dynamometer. Patients were seated with the wrist in a neutral position and the elbow flexed 90°. In case a subject was unable to sit, grip strength was assessed lying at 30° in bed with the elbows supported. Data on grip strength was missing for 21 patients.

### Analytical approach

LoS was defined as the number of days from admission to discharge (or death). For the analyses regarding LoS, continuous ADL score was used. Walking speed performance was categorised into unable to perform the test, <0.8 m/s, and ≥0.8 m/s [17]. Grip strength categories were: unable to perform the test at admission, <21 kg in women or <37 kg in men, and ≥21 kg in women or ≥37 kg in men [18]. New institutionalisation comprised subjects who lived at home before admission and were transferred to a residence for older people or to a nursing home at discharge. For the analyses regarding mortality and institutionalisation, ADL total dependency (6/6 dependencies) was used and only two categories of walking speed performance and grip strength were considered (able vs. unable to perform the test), due to the limited number of deaths and institutionalisations. Participants were considered ‘unable’ to perform a test, if they could not follow instructions to complete the test, if they could not complete the test because of their health condition (i.e. drowsiness or severe cognitive impairment), or in the case of walking speed, if the researchers considered it unsafe to perform the test.

### Statistical analyses

Descriptive data are presented as mean ± SD, median (first to third quartile) or percentage, where appropriate. Multiple linear regression was used to predict LoS. Models were adjusted for centre, gender, age, and type of admission. Model validity was tested for the linearity of the regression function, the constancy of variance, and the independence and normality of error terms. LoS was natural log-transformed, because the untransformed variable was highly skewed. The approximate interpretation of B is: Length of hospital stay changes by 100*B percent for a one unit increase in the independent variable. Change in average length of stay was calculated as follows: B * (mean length of stay) = B * 11.17 days. Subsequent analyses included all significant predictors of LoS in a multivariate regression model with forward selection procedure and forced retention of centre, gender, age, and type of admission.

Multiple logistic regression was used to predict in-hospital mortality and institutionalisation. Models were adjusted for centre and age. Subsequent analyses included all significant predictors of in-hospital mortality or institutionalisation in a multivariate regression model with forward selection procedure and forced retention of centre and age. The Nagelkerke R^2^ statistic [19] was used to assess the amount of variation in in-hospital mortality and institutionalisation, ‘explained’ by the variables.

Subjects who died in-hospital were excluded from the analyses of institutionalisation. Of patients who were hospitalised more than once during the enrolment period, only data concerning the first hospitalisation was considered.

All analyses were performed using SPSS software, version 19.0 (SPSS Inc., Chicago, IL). Statistical significance was indicated by a P value <0.05; all P values were two-tailed.

## Results

### Study population

In total, 1123 participants were enrolled in the CRIME study. Age ranged between 65 and 102 years (mean 81.5±7.4 y). Slightly more than half of the participants (56%, N = 629) were female. Subjects had a mean BMI of 26±5 kg/m^2^, 4% (N = 41) were underweight (BMI<18.5 kg/m^2^). Functional status fluctuated between both ends of the spectrum, with 36% (N = 404) having no ADL impairments and 22% (N = 252) being totally dependent in ADL. Mean walking speed performance was 0.65±0.25 m/s. Grip strength performers had a mean of 20±9 kg. Further characteristics of the study population are described in Table 1.

**Table 1:** Characteristics of the study population (N = 1223).

Variable	Value
***Socio-demographic factors***	
Age (years), mean ± SD	81.5±7.4
Gender (female), % (N)	56 (629)
Elective admission, % (N)	49 (551)
Living alone, % (N)	25 (279)
***Medical history***	
No of drugs before admission, mean ± SD	6±3
≥2 hospital admissions during the last year, % (N)	19 (216)
***Medical diagnoses***	
Comorbidity score, median (IQR)	5 (3–6)
Ischemic heart disease, % (N)	32 (356)
Heart failure, % (N)	27 (307)
Cerebrovascular accident, % (N)	21 (236)
Parkinson’s disease, % (N)	6 (68)
Dementia (Alzheimer or other), % (N)	21 (230)
Diabetes mellitus, % (N)	30 (333)
Metastasized cancer, % (N)	4 (47)
Renal failure or dialysis, % (N)	26 (286)
Infection, % (N)	11 (119)
***Clinical conditions***	
Falls at home during the last year, % (N)	25 (278)
Pain, % (N)	52 (589)
Pressure ulcers, % (N)	5 (57)
Urinary incontinence or catheter, % (N)	42 (468)
Faecal incontinence, % (N)	14 (157)
Malnutrition (BMI <18.5 kg/m^2^), % (N)	4 (41)
***Cognitive and affective status***	
30 item Mini Mental State Examination category, % (N)	
Unable	17 (186)
<24	41 (460)
≥24	37 (415)
15 item Geriatric Depression Scale, median (IQR)	4 (2–7)
***Functional status and physical performance***	
ADL score (/6), median (IQR)	1 (0–5)
ADL total dependency, % (N)	22 (252)
Walking speed category, % (N)	
Unable	54 (603)
<0.8 m/s	34 (381)
≥0.8 m/s	12 (139)
Grip strength category, % (N)	
Unable	27 (306)
<21 kg ♀, <37 kg ♂	62 (700)
≥21 kg ♀, ≥37 kg ♂	9 (96)

SD = Standard deviation; IQR = Interquartile range; BMI = Body Mass Index;

ADL = Activities of Daily Living.

### Length of stay

LoS varied from one to 65 days, with 75% of subjects staying in hospital between seven and fourteen days (median 10 days). Factors predictive of LoS in initial analyses were number of drugs before admission, more than one hospital admission during the last year, comorbidity score, presence of heart failure, metastasized cancer, renal failure of infection, falls at home during the last year, pain, ADL score/total dependency and walking speed category/inability (S1 Table). Subsequent multivariate analyses, which included these significant variables, identified number of drugs before admission, metastasized cancer, renal failure or dialysis, infection, falls at home during the last year, pain, and walking speed category as independent predictors of LoS (Table 2).

**Table 2:** Independent predictors of length of hospital stay.

	Variables	B (CI_95_)
**Length of hospital stay**	No of drugs before admission (/3)	0.04 (0.01–0.07)
	Metastasized cancer	0.31 (0.15–0.47)
	Renal failure or dialysis	0.16 (0.08–0.23)
	Infection	0.16 (0.05–0.27)
	Falls at home during the last year	0.12 (0.04–0.19)
	Pain	0.07 (0.00–0.14)
	Walking speed category	−0.07 (−0.12– −0.01)

Analyses of length of hospital stay included number of drugs before admission, more than one hospital admission during the last year, comorbidity score, presence of heart failure, metastasized cancer, renal failure of infection, falls at home during the last year, pain, ADL score and walking speed category in a multivariate regression model with forward selection procedure and forced retention of centre, gender, age, and type of admission.

CI_95_ = 95% confidence interval; ADL = Activities of Daily Living; BMI = Body Mass Index.

### In-hospital mortality

Forty one (4%) patients died in hospital. Factors predictive of in-hospital mortality in initial analyses were age, infection, falls at home during the last year, pressure ulcers, urinary or faecal incontinence, MMSE category, ADL score/total dependency, walking speed inability, and grip strength inability (S2 Table). Subsequent multivariate analyses, which included these significant variables, identified ADL total dependency and grip strength inability as independent predictors of in-hospital mortality (Table 3). This multivariate model explained 28.5% of the variability in in-hospital mortality.

**Table 3:** Independent predictors of in-hospital mortality and institutionalisation.

	Variables	OR (CI_95_)
**In-hospital mortality**	ADL total dependency	3.8 (1.5–9.8)
	Grip strength inability	5.6 (2.0–16)
**Institutionalisation**	Malnutrition (BMI <18.5 kg/m^2^)	7.6 (2.0–29)
	ADL total dependency	8.0 (2.8–23)

Analyses of in-hospital mortality included infection, falls at home during the last year, pressure ulcers, urinary or faecal incontinence, MMSE category, ADL total dependency, walking speed inability, and grip strength inability with forward selection procedure and forced retention of centre and age.

Analyses of institutionalisation included cerebrovascular accident, dementia, metastasized cancer, pain, urinary incontinence, malnutrition, MMSE category, ADL total dependency, walking speed inability, and grip strength inability with forward selection procedure and forced retention of centre and age. Subjects who died in-hospital were excluded from the analyses.

OR = Odds ratio, CI_95_ = 95% confidence interval; ADL = Activities of Daily Living; BMI = Body Mass Index.

### Institutionalisation

Thirty seven patients (3%) were newly institutionalised after discharge. Factors predictive of institutionalisation in initial analyses were age, cerebrovascular accident, dementia, metastasized cancer, pain, urinary incontinence, malnutrition, MMSE category, ADL score/total dependency, walking speed inability, and grip strength inability (S3 Table). Subsequent multivariate analyses, which included these significant variables, identified malnutrition and ADL total dependency as independent predictors of institutionalisation (Table 3). This model explained 32.9% of the variability in institutionalisation.

## Discussion

In this multicentre study, we aimed to identify factors related to older patients’ clinical, nutritional, functional and socio-demographic profiles at admission to an acute care ward that can predict LoS, in-hospital mortality, and institutionalisation.

The observed median LoS of 10 days in this study is in line with previous reports of multicentre Italian and European studies [20], [21]. The number of drugs before admission was predictive of longer LoS. This corresponds with the reported association between polypharmacy and poor hospital outcomes [4], [5]. Along with other studies, we found the occurrence of infection [22], having a history of falls at home [1], [23], and slower walking speed [6], [7], [23]–[25] were associated with longer LoS. The predictive value of falls may not only be explained by poor walking mobility, as the association between falls and LoS persists after adjustment for walking speed in the multivariate analyses. The presence of pain has not been reported as a predictor of LoS in the acute care setting previously, but this finding is consistent with a reported association between pain perception and LoS in older patients with hip fracture [26].

The observed percentage of in-hospital mortality (4%) and new institutionalisation (3%) in this study are in line with previous reports of multicentre Italian and European studies [20], [21], [27]. The presence of malnutrition and ADL total dependency both led to an eight-fold increase in institutionalisation rate. Previous studies likewise found malnutrition to be independently predictive of institutionalisation [2], [28], [29]. This may be explained in part by the fact that malnutrition in older hospitalised patients is a result of several medical and social conditions that chronically affect them, rather than an indicator of an acute illness [28]. In agreement with other studies, we found that ADL total dependency was independently predictive of in-hospital mortality [4], [21], [28] and institutionalisation [2], [21]. Inability to perform the grip strength test led to a five-fold increase in mortality rate. Grip strength is both a marker of functional and nutritional status [2].

The added value of physical performance measurements over self-reported measurements has been questioned [6], [7], [30], [31]. In our study, walking speed was a better predictor for LoS than ADL score. Likewise, other studies found the association between functional status and LoS less discriminating compared with walking speed [6], [7]. Self-reported functional status measures, such as ADL score, are not designed to capture the entire range of function in older adults [6] and may display ceiling effects [30]. Therefore, in older persons with few ADL dependencies, physical performance measures might be more valuable [30]. On the other hand, ADL total dependency as a measure of functional status was more discriminating for in-hospital mortality and institutionalisation than walking speed performance. First, the walking speed test displayed a floor effect in the weakest patients. Only few subjects who died in-hospital or who were newly institutionalised could perform the walking speed test (N = 1 and 4, respectively) at admission. Second, functional status encompasses physical function, cognitive status, and incontinence; these all have predictive value for institutionalisation.

This study has both strengths and limitations. Overall, 1541 eligible patients were screened and 1123 (73%) agreed to participate in the study [9]. These non-participants might have had poorer hospital outcomes than participants and hence might bias our results. Despite the multicentre design of our study, findings may not be representative for all older patients admitted to an acute care ward, because the centres involved were exclusively Italian facilities and they were not randomly chosen. Outcomes studied (in-hospital mortality, new institutionalisation and especially LoS) can be highly influenced by the characteristics of the hospital and their staff. However, the aim of the present study was to assess factors related to older patients’ profiles rather than organisational characteristics. In addition, analyses were adjusted for ‘centre’ in order to take into account the effect of centres’ characteristics on study outcomes. One strength of this study is that data was prospectively collected on multifaceted variables, which are otherwise unavailable in administrative datasets and in many chart-based studies. It is interesting to note that routinely available socio-demographic factors were not predictive of any hospital outcome. Similar findings have been reported by other studies [1], [4], [23]. We believe that such a multi-dimensional approach to identify predictors of hospital outcomes in older in-patients might add value to the organisation of healthcare in comparison with single-component assessments.

The ageing ratio in Italy has been increasing over the past decades, with 146.5 persons aged 65 and over compared to 100 persons aged 0–14 in 2010 [32]. Mean life expectancy at the age of 65 has reached 22.1 years for women (with 10 healthy life years) and 18.3 years for men (with 10.2 healthy life years) [33]. In 2010, over 10 million persons were admitted to acute care in Italy, of which 40% were aged 65 and older [34]. There were 287 acute care beds in hospitals per 100 000 inhabitants [35]. This number has decreased over the last years, which may be explained by the shift in resources from the hospital to the community through the realisation of integrated home care and residential health care facilities. The number of facilities is increasing over the years with 6153 residential health care facilities in 2010 [36], of which 3749 provided health care for older persons [37]. However, Italy still has a low number of residential beds for older persons compared to other Western countries. This may be due to the Italian family structure, which traditionally considers care of older people as a responsibility of the family [38] and it may explain the low rate of institutionalisation observed in our study sample.

One of Italy’s challenges to satisfy the needs of frail older people is the presence of Geriatric Evaluation Units in Health agencies to perform comprehensive geriatric assessments [38]. If older patients at risk of poor hospital outcomes could be identified at admission, than appropriate resources could be made available during hospitalisation, enabling a more effective healthcare service [1]. This can be achieved by implementing a comprehensive geriatric assessment at admission to evaluate physical performance, functional dependency, nutritional status, polypharmacy, and falls. Our results may therefore have not only important implications for the organisation and quality of clinical practice but also for its economic aspects.

Based on our results, in addition to nutrition optimization, we encourage exercise and physical therapy interventions, which may help to prevent falls and to improve physical performance. We endorse that patients’ drug use is carefully reviewed in order to discontinue potentially inappropriate medication [20], particularly by withdrawing drugs of little added value and limiting those related to falling.

Our results confirm that not only diseases, but also multifaceted aspects of ageing such as physical function and malnutrition are strong predictors of hospital outcomes and suggest that these variables should be systematically recorded [21], [39].

## Supporting Information

S1 Table **Predictors of length of stay.** Data reported are from different linear regression models predicting length of stay (natural log-transformed). Each model included centre, age, gender, and type of admission as covariates. The approximate interpretation of B is: Length of hospital stay changes by 100*B percent for a one unit increase in the independent variable. Change in average length of stay was calculated as follows: B * (mean length of stay) = B * 11.17 days. B = regression coefficient LN(length of stay); CI_95_ = 95% confidence interval; LoS = length of stay; BMI = Body Mass Index; MMSE = Mini Mental State Examination; ADL = Activities of Daily Living *** Statistical significance was indicated by a P value <0.05.
(DOCX)

S2 Table **Predictors of in-hospital mortality.** Data reported are from different logistic regression models predicting in-hospital mortality. Each model included centre and age as covariates. OR = Odds ratio, CI_95_ = 95% confidence interval; BMI = Body Mass Index; MMSE = Mini Mental State Examination; ADL = Activities of Daily Living *** Statistical significance was indicated by a P value <0.05.
(DOCX)

S3 Table **Predictors of institutionalisation.** Data reported are from different logistic regression models predicting new institutionalisation. Each model included centre and age as covariates. Subjects who died in-hospital were excluded from the analyses. OR = Odds ratio, CI_95_ = 95% confidence interval; BMI = Body Mass Index; MMSE = Mini Mental State Examination; ADL = Activities of Daily Living *** Statistical significance was indicated by a P value <0.05.
(DOCX)
