# Supplementary material for: A Multicomponent Approach to Identify Predictors of Hospital Outcomes in Older In-Patients: A Multicentre, Observational Study
Source: PLoS One. 2014 Dec 26;9(12):e115413. doi: 10.1371/journal.pone.0115413 (PMC4277310; doi:10.1371/journal.pone.0115413)
Supplement: S1 Table — Predictors of length of stay. Data reported are from different linear regression models predicting length of stay (natural log-transformed). Each model included centre, age, gender, and type of admission as covariates. The approximate interpretation of B is: Length of hospital stay changes by 100*B percent for a one unit increase in the independent variable. Change in average length of stay was calculated as follows: B * (mean length of stay) = B * 11.17 days. B = regression coefficient LN(length of stay); CI95 = 95% confidence interval; LoS = length of stay; BMI = Body Mass Index; MMSE = Mini Mental State Examination; ADL = Activities of Daily Living *** Statistical significance was indicated by a P value <0.05. (DOCX) [file pone.0115413.s001.docx]

Table S1. Predictors of length of stay.

| **Variable** | **Β (CI_95_)** | **P** | **Change in average LoS (days)** |
| --- | --- | --- | --- |
| ***Socio-demographic factors*** |  |  |  |
| Age (years) | 0.00 (-0.00 – 0.01) | 0.10 | 0.04 |
| Gender (female) | 0.01 (-0.05 – 0.08) | 0.69 | 0.15 |
| Elective admission | -0.06 (-0.15 – 0.02) | 0.15 | -0.70 |
| Living alone | 0.03 (-0.05 – 0.10) | 0.49 | 0.30 |
| ***Medical history*** |  |  |  |
| No of drugs before admission (/3)*** | 0.07 (0.04 – 0.10) | <0.001 | 0.74 |
| ≥2 hospital admissions during the last year*** | 0.09 (0.01 – 0.18) | 0.04 | 1.01 |
| ***Medical diagnoses*** |  |  |  |
| Comorbidity score*** | 0.03 (0.01 – 0.05) | <0.001 | 0.34 |
| Ischemic heart disease | 0.04 (-0.03 – 0.11) | 0.28 | 0.42 |
| Heart failure | 0.07 (-0.01 – 0.14) | 0.09 | 0.75 |
| Cerebrovascular accident | 0.06 (-0.14 – 0.02) | 0.16 | 0.65 |
| Parkinson's disease | -0.02 (-0.15 – 0.11) | 0.77 | -0.22 |
| Dementia (Alzheimer or other) | -0.01 (-0.10 – 0.08) | 0.80 | -0.12 |
| Diabetes mellitus | 0.05 (-0.02 – 0.12) | 0.17 | 0.55 |
| Metastasized cancer*** | 0.31 (0.15 – 0.47) | <0.001 | 3.48 |
| Renal failure or dialysis*** | 0.18 (0.11 – 0.26) | <0.001 | 2.02 |
| Infection*** | 0.18 (0.07 – 0.29) | 0.002 | 2.00 |
| ***Clinical conditions*** |  |  |  |
| Falls at home during the last year*** | 0.12 (0.05 – 0.20) | 0.002 | 1.35 |
| Pain*** | 0.11 (0.04 – 0.18) | 0.002 | 1.21 |
| Pressure ulcers | 0.03 (-0.12 – 0.18) | 0.68 | 0.35 |
| Urinary incontinence or catheter | 0.07 (-0.00 – 0.14) | 0.06 | 0.77 |
| Faecal incontinence | 0.10 (0.00 – 0.20) | 0.05 | 1.12 |
| Malnutrition (BMI <18.5 kg/m²) | -0.11 (-0.28 – 0.06) | 0.20 | -1.25 |
| ***Cognitive and affective status*** |  |  |  |
| 30 item MMSE category | -0.04 (-0.10 – 0.01) | 0.09 | -0.49 |
| 15 item Geriatric Depression Scale | 0.01 (-0.01 – 0.02) | 0.43 | 0.06 |
| ***Functional status and physical performance*** | |  |  |
| ADL score*** | 0.02 (0.00 – 0.04) | 0.02 | 0.23 |
| ADL total dependency | 0.09 (0.00 – 0.18) | 0.05 | 1.01 |
| Walking speed category*** | -0.10 (-0.15 – -0.04) | <0.001 | -1.06 |
| Walking speed inability*** | 0.12 (0.05 – 0.20) | 0.001 | 1.34 |
| Grip strength category | -0.02 (-0.09 – 0.04) | 0.53 | -0.23 |
| Grip strength inability | -0.04 (-0.12 – 0.04) | 0.32 | -0.45 |

Data reported are from different linear regression models predicting length of stay (natural log-transformed). Each model included centre, age, gender, and type of admission as covariates.

The approximate interpretation of B is: Length of hospital stay changes by 100*B percent for a one unit increase in the independent variable.

Change in average length of stay was calculated as follows: B * (mean length of stay) = B * 11.17 days.

B = regression coefficient LN(length of stay); CI_95_ = 95% confidence interval; LoS= length of stay; BMI = Body Mass Index; MMSE = Mini Mental State Examination; ADL = Activities of Daily Living

*** Statistical significance was indicated by a P value <0.05
