# Supplementary material for: A Multicomponent Approach to Identify Predictors of Hospital Outcomes in Older In-Patients: A Multicentre, Observational Study
Source: PLoS One. 2014 Dec 26;9(12):e115413. doi: 10.1371/journal.pone.0115413 (PMC4277310; doi:10.1371/journal.pone.0115413)
Supplement: S2 Table — Predictors of in-hospital mortality. Data reported are from different logistic regression models predicting in-hospital mortality. Each model included centre and age as covariates. OR = Odds ratio, CI95 = 95% confidence interval; BMI = Body Mass Index; MMSE = Mini Mental State Examination; ADL = Activities of Daily Living *** Statistical significance was indicated by a P value <0.05. (DOCX) [file pone.0115413.s002.docx]

Table S2. Predictors of in-hospital mortality.

| **Variable** | **OR (CI_95_)** | **P** |  |
| --- | --- | --- | --- |
| ***Socio-demographic factors*** |  |  |  |
| Age (years)*** | 1.10 (1.05 – 1.17) | <0.001 |  |
| Gender (female) | 0.72 (0.37 – 1.39) | 0.33 |  |
| Elective admission | 0.54 (0.21 – 1.38) | 0.20 |  |
| Living alone | 1.09 (0.50 – 2.35) | 0.83 |  |
| ***Medical history*** |  |  |  |
| No of drugs before admission (/3) | 1.09 (0.80 – 1.48) | 0.60 |  |
| ≥2 hospital admissions during the last year | 0.70 (0.31 – 1.56) | 0.38 |  |
| ***Medical diagnoses*** |  |  |  |
| Comorbidity score | 1.03 (0.89 – 1.19) | 0.71 |  |
| Ischemic heart disease | 1.14 (0.58 – 2.23) | 0.70 |  |
| Heart failure | 1.34 (0.66 – 2.75) | 0.42 |  |
| Cerebrovascular accident | 0.83 (0.40 – 1.72) | 0.62 |  |
| Parkinson's disease | 0.59 (0.14 – 2.58) | 0.48 |  |
| Dementia (Alzheimer or other) | 1.17 (0.59 – 2.33) | 0.65 |  |
| Diabetes mellitus | 0.89 (0.42 – 1.90) | 0.77 |  |
| Metastasized cancer | 0.00 (0.00 – .) | 1.00 |  |
| Renal failure or dialysis | 1.79 (0.91 – 3.50) | 0.09 |  |
| Infection*** | 2.69 (1.21 – 5.99) | 0.02 |  |
| ***Clinical conditions*** |  |  |  |
| Falls at home during the last year*** | 0.39 (0.16 – 0.97) | 0.04 |  |
| Pain | 1.17 (0.55 – 2.55) | 0.69 |  |
| Pressure ulcers*** | 4.93 (1.89 – 12.9) | 0.001 |  |
| Urinary incontinence or catheter*** | 4.12 (1.64 – 10.3) | 0.003 |  |
| Faecal incontinence*** | 2.92 (1.45 – 5.90) | 0.003 |  |
| Malnutrition (BMI <18.5 kg/m²) | 2.26 (0.77 – 6.65) | 0.14 |  |
| ***Cognitive and affective status*** |  |  |  |
| 30 item MMSE category*** | 0.42 (0.24 – 0.75) | 0.003 |  |
| 15 item Geriatric Depression Scale | 1.09 (0.83 – 1.44) | 0.53 |  |
| ***Functional status and physical performance*** |  |  |  |
| ADL score*** | 1.08 (1.02 – 1.14) | 0.005 |  |
| ADL total dependency*** | 5.60 (2.45 – 12.8) | <0.001 |  |
| Walking speed inability*** | 14.8 (1.91 – 114) | 0.010 |  |
| Grip strength inability*** | 8.03 (3.17 – 20.3) | <0.001 |  |

Data reported are from different logistic regression models predicting in-hospital mortality. Each model included centre and age as covariates.

OR = Odds ratio, CI_95_ = 95% confidence interval; BMI = Body Mass Index; MMSE = Mini Mental State Examination; ADL = Activities of Daily Living

*** Statistical significance was indicated by a P value <0.05
