# Supplementary material for: A Multicomponent Approach to Identify Predictors of Hospital Outcomes in Older In-Patients: A Multicentre, Observational Study
Source: PLoS One. 2014 Dec 26;9(12):e115413. doi: 10.1371/journal.pone.0115413 (PMC4277310; doi:10.1371/journal.pone.0115413)
Supplement: S3 Table — Predictors of institutionalisation. Data reported are from different logistic regression models predicting new institutionalisation. Each model included centre and age as covariates. Subjects who died in-hospital were excluded from the analyses. OR = Odds ratio, CI95 = 95% confidence interval; BMI = Body Mass Index; MMSE = Mini Mental State Examination; ADL = Activities of Daily Living *** Statistical significance was indicated by a P value <0.05. (DOCX) [file pone.0115413.s003.docx]

Table S3. Predictors of institutionalisation.

| **Variable** |  | **OR (CI_95_)** | **P** |
| --- | --- | --- | --- |
| ***Socio-demographic factors*** |  |  |  |
| Age (years)*** |  | 1.10 (1.04 – 1.16) | 0.001 |
| Gender (female) |  | 1.25 (0.61 – 2.57) | 0.54 |
| Elective admission |  | 1.61 (0.60 – 4.32) | 0.34 |
| Living alone |  | 0.96 (0.43 – 2.14) | 0.93 |
| ***Medical history*** |  |  |  |
| No of drugs before admission (/3) |  | 1.03 (0.73 – 1.43) | 0.88 |
| ≥2 hospital admissions during the last year |  | 1.69 (0.79 – 3.60) | 0.18 |
| ***Medical diagnoses*** |  |  |  |
| Comorbidity score |  | 1.05 (0.91 – 1.22) | 0.53 |
| Ischemic heart disease |  | 0.72 (0.34 – 1.52) | 0.39 |
| Heart failure |  | 1.09 (0.50 – 2.37) | 0.82 |
| Cerebrovascular accident*** |  | 2.10 (1.03 – 4.29) | 0.04 |
| Parkinson's disease |  | 1.83 (0.59 – 5.74) | 0.30 |
| Dementia (Alzheimer or other)*** |  | 3.47 (1.64 – 7.74) | 0.001 |
| Diabetes mellitus |  | 1.08 (0.51 – 2.28) | 0.85 |
| Metastasized cancer*** |  | 3.10 (1.14 – 8.48) | 0.03 |
| Renal failure or dialysis |  | 1.13 (0.54 – 2.36) | 0.75 |
| Infection |  | 1.90 (0.84 – 4.29) | 0.13 |
| ***Clinical conditions*** |  |  |  |
| Falls at home during the last year |  | 1.43 (0.70 – 2.93) | 0.33 |
| Pain*** |  | 2.47 (1.04 – 5.86) | 0.04 |
| Pressure ulcers |  | 2.74 (0.54 – 13.9) | 0.22 |
| Urinary incontinence or catheter*** |  | 2.47 (1.12 – 5.45) | 0.03 |
| Faecal incontinence |  | 2.28 (0.98 – 5.28) | 0.06 |
| Malnutrition (BMI <18.5 kg/m²)*** |  | 4.51 (1.42 – 14.34) | 0.01 |
| ***Cognitive and affective status*** |  |  |  |
| 30 item MMSE category*** |  | 0.33 (0.18 – 0.59) | < 0.001 |
| 15 item Geriatric Depression Scale |  | 1.10 (0.95 – 1.28) | 0.21 |
| ***Functional status and physical performance*** |  |  |  |
| ADL score*** |  | 1.60 (1.27 – 2.01) | < 0.001 |
| ADL total dependency*** |  | 5.65 (2.44 – 13.1) | < 0.001 |
| Walking speed inability*** |  | 5.34 (1.69 – 16.9) | 0.004 |
| Grip strength inability*** |  | 3.22 (1.35 – 7.70) | 0.01 |

Data reported are from different logistic regression models predicting new institutionalisation. Each model included centre and age as covariates. Subjects who died in-hospital were excluded from the analyses.

OR = Odds ratio, CI_95_ = 95% confidence interval; BMI = Body Mass Index; MMSE = Mini Mental State Examination; ADL = Activities of Daily Living

*** Statistical significance was indicated by a P value <0.05
